# High CFP score indicates poor prognosis and chemoradiotherapy response in LARC patients

**DOI:** 10.1186/s12935-021-01903-1

**Published:** 2021-04-13

**Authors:** Siyi Lu, Zhenzhen Liu, Bingyan Wang, Fei Li, Yan Meng, Junwei Wang, Yuxia Wang, Hao Wang, Xin Zhou, Wei Fu

**Affiliations:** 1 Department of General Surgery, Peking University Third Hospital, Beijing, 100191 China; 2 Department of Radiotherapy, Peking University Third Hospital, Beijing, 100191 China

**Keywords:** Rectal cancer, CFP, Prognosis, Tumor regression grade

## Abstract

**Background:**

Preoperative tumor markers, inflammation, and nutritional status are considered important predictors of prognosis and tumor response in locally advanced rectal cancer (LARC) patients. This study aims to explore the prognostic and predictive role of carcinoembryonic antigen (CEA), the Fibrinogen-Albumin Ratio Index (FARI), the Prognostic Nutritional Index (PNI) in LARC patients and compared them with a novel combined CEA-FARI-PNI (CFP) scoring system.

**Methods:**

A total of 138 LARC patients undergoing radical surgery following neoadjuvant chemoradiotherapy (NCRT) between January 2012 and March 2019 were enrolled. The X-tile program was used to determine the optimal cut-off values of CEA, FARI, and PNI, and CFP scoring system was constructed accordingly. The prognostic ability of these factors was assessed by the time-dependent receiver operating characteristic (ROC) curve, Kaplan–Meier, Cox regression, and logistic regression. Nomogram was established to evaluate the predictive role of these factors in tumor response.

**Results:**

The optimal cut-off values of CEA, FARI, and PNI were 5.15 ng/l, 10.56%, and 42.25 g/L, respectively. The time-dependent ROC curve showed that compared to CEA, FARI, and PNI, CFP showed stable predictive efficacy for overall survival (OS) and disease-free survival (DFS). In multivariate analysis, CFP was the only factor that could independently predict OS (HR = 8.117, p = 0.001) and DFS (HR = 4.994, p < 0.001). Moreover, high CFP (OR = 3.693, p = 0.002) was also an independent risk factor of poor response. The area under the ROC curve (AUC) of the nomograms for predicting tumor response was better including CFP (0.717) than without CFP (0.656) (p < 0.05).

**Conclusions:**

The CFP score was a more reliable marker for predicting OS, DFS, and NCRT efficacy in LARC patients, and the score could apparently improve predicted efficacy of the nomogram.

**Supplementary Information:**

The online version contains supplementary material available at 10.1186/s12935-021-01903-1.

## Introduction

Colorectal cancer is one of the most common cancers worldwide and is the second leading cause of cancer-related deaths [1]. Rectal cancer accounts for nearly 30% of all colorectal cancers [2]. Currently, preoperative neoadjuvant chemoradiotherapy (NCRT) is thought to improve local pelvic control and decrease the incidence of local relapse and has become the standard regimen for locally advanced rectal cancer (LARC) patients. Approximately 50–60% of patients are downstaged after NCRT, and 10–30% will achieve a pathological complete response [3]. Although standard treatments are available for these patients, including NCRT, total mesorectal excision (TME), and adjuvant chemotherapy, local relapse and distant metastasis remain the leading problems of LARC [4, 5]. Hence, more economical and feasible preoperative clinical biomarkers are needed to stratify patients with high-risk status and to guide tailored treatment.

Carcinoembryonic antigen (CEA) is widely used as a prognostic marker for colorectal cancer patients worldwide. Previous studies [6–8] have shown that serum CEA was associated with tumor response and prognosis in rectal cancer patients undergoing curative excision. Moreover, the preoperative CEA level may play a determinant role in the early detection of recurrent disease during follow-up after the TME procedure.

The cancer-related systemic inflammatory response and alterations in nutritional status have been identified as some of the most critical hallmarks of solid tumors [9, 10]. Inflammation may facilitate the proliferation and distance seeding of malignant cells, leading to tumor progression and metastasis, inhibiting adaptive immunity, and even altering tumor sensitivity to NCRT [11–13]. Meanwhile, malnutrition is associated with decreased immune function [14], weakened physical status[15], and poor NCRT outcomes [16], leading to increased mortality among cancer patients. The fibrinogen-to-albumin index (FARI) is considered an essential biomarker that reflects both systemic inflammatory status and nutritional status, and several studies have reported that FARI is closely related to the prognosis of various cancers, such as breast cancer [17], esophageal cancer [18], and gastric cancer [19]. Our previous findings have shown similar results in LARC patients undergoing TME following NCRT, and we have found that FARI is associated with tumor response [20]. The prognostic nutritional index (PNI), based on the albumin level and lymphocyte count, is another widely used biomarker that combines inflammatory and nutritional parameters. Okugawa et al. [21] analyzed 114 rectal cancer patients who underwent NCRT and demonstrated that PNI could predict survival and tumor response.

Since CEA [7], FARI [20] and PNI [21] have all been found to serve as indicators of the prognosis and tumor response of LARC patients, we constructed a combination of these markers and investigated the prognostic and predictive role of the combination (CEA-FARI-PNI, CFP) in 138 LARC patients undergoing radical surgery following NCRT and compared its prognosis predicted efficacy with CEA, FARI and PNI. Here, we reported a novel CFP scoring system could independently predict survival of LARC patients and precisely identify different NCRT response among LARC patients.

## Methods

### Study population

A total of 138 consecutive LARC (cTNM stage II or stage III) patients from Peking University Third Hospital between March 2012 and March 2019 were ultimately enrolled and followed. Ethical approval was obtained from the Ethics Committee of Peking University Third Hospital (IRB00006761-M2019387), and this study adhered to the tenets of the Declaration of Helsinki. The inclusion criteria were as follows: (1) diagnosis of LARC through preoperative MR and CT and received NCRT followed by radical surgery; (2) diagnosis of adenocarcinoma via postoperative histopathologically; (3) complete resection without positive tumor margins; and (4) complete inpatient data, including preoperative complete blood counts and follow-up data. The exclusion criteria were as follows: (1) anti-immunosuppressive or anti-inflammatory treatments; (2) autoimmune disease, hematological disease, and acute infection; 3) the presence of other cancers in addition to rectal adenocarcinoma; and 4) emergency surgery for obstruction or perforation of the rectum.

### Clinicopathological data and definitions

Hematological examinations included routine blood examination, liver function tests, coagulation tests, and CEA measurements. All blood specimens were tested in the laboratory of our hospital within two weeks before the operation. PNI and FARI were defined as follows: PNI = albumin (g/L) + 5 × lymphocyte count (10^9^/L); FARI = the ratio of fibrinogen (g/L) to albumin (g/L) × 100%. The AJCC-TRG definitions were as follows: TRG0, no sign of tumor cells; TRG1, single tumor cell or small groups of tumor cells can be detected; TRG2, residual cancer with a desmoplastic response (mild regression); and TRG3, no regression. In this study, TRG0-1 was defined as a good response, while TRG2-3 was defined as a poor response.

### Treatment and follow-up

All eligible patients received radiation according to institutional protocols. Oral capecitabine at a dose of 1,650 mg/m^2^ per daily was administered concurrently with radiotherapy. Six to 9 weeks after the end of chemoradiotherapy, the LARC patients underwent curative TME, which was performed by four experienced colorectal surgeons at Peking University Third Hospital. Patients were followed-up at 1 and 3 months after surgery and every 6 months thereafter. Abdominal and pelvic contrast-enhanced CT or MRI scans and CEA levels were routinely performed every 6 months for 2 years and then once every year for a total of 3 years at each follow-up. Colonoscopy was conducted within 1 year after surgery and then repeated every 2–3 years. The presence of new lesions revealed by biopsy or imaging was deemed tumor recurrence. Appropriate treatment, such as repeated surgery, systemic chemotherapy, radiofrequency ablation, or RT, was performed for patients with tumor recurrence. The period from radical surgery to death was defined as OS, and the period from radical surgery to any local or distant recurrence was defined as DFS.

### Construction of the novel prognostic scoring system

**Fig. 1:**
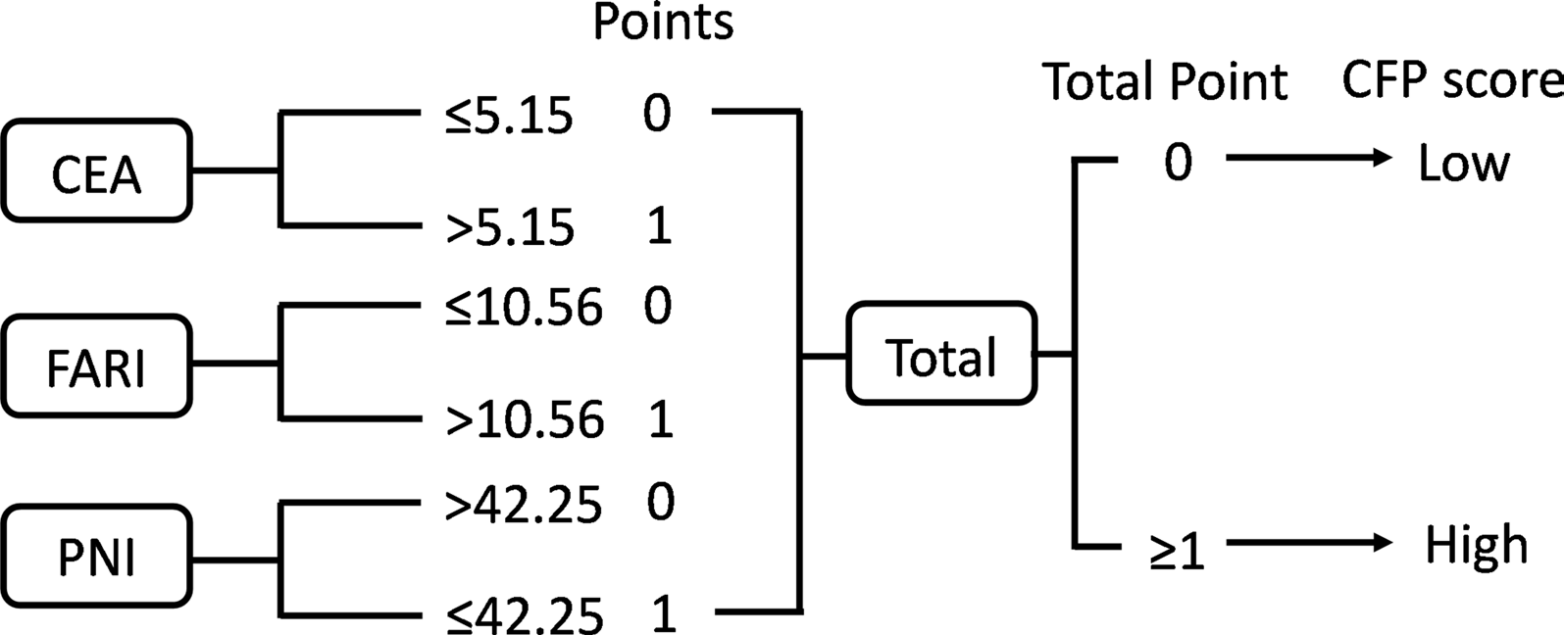
The detailed definition of the CFP score

A novel tumor marker, inflammation- and nutrition-based prognostic score, CFP (a combination of CEA, FARI, and PNI), was constructed in this study. CEA levels and FARI scores lower or higher than the cut-off values were considered 0 and 1 point, respectively, while levels of PNI higher or lower than the cut-off values were considered 0 and 1 point, respectively. Total scores of 0 and ≥ 1 were defined as low and high CFP scores, respectively (Fig. 1).

### Statistical analysis

The X-tile program was used to determine the optimal cut-off values of CEA, FARI, and PNI. The time-dependent ROC analysis to compare the prognostic values of the markers for DFS and OS was performed by ‘timeROC’ packages in R version 3.5.2. Normality was tested using the Shapiro–Wilk test. Independent sample t-tests, chi-square tests, and Fisher’s exact tests were used to analyze the correlation between the CFP score and clinicopathological parameters. Kaplan–Meier curves of patients stratified by CEA, FARI, PNI, and CFP values were generated for DFS and OS, and the log-rank test was used to calculate p values. Univariate and multivariate analyses of the Cox proportional hazards model were used to determine the factors that may correlate with DFS and OS, while univariate and multivariate analyses of logistic regression were used to determine the factors that may be associated with TRG. Potential risk factors (P < 0.1) were adopted for multivariate analysis with the backward stepwise method following univariate analysis. According to the multivariate analysis results of logistic regression, a prognostic nomogram for predicting the TRG of LARC patients was established, and the AUC and calibration curve verified its predictive ability. The logistic regression nomogram was established by the ‘rms’ package in R. All statistical analyses were carried out by SPSS Statistics 19.0 (IBM Corporation, Armonk, NY, USA). A P value < 0.05 was recognized as statistically significant.

## Results

### Patient characteristics

Among the 138 LARC patients enrolled (Additional file 1: Figure S1), male patients accounted for the majority (72.5%), and the median age was 60 years (range 53–69). A total of 118 (85.5%) patients had tumors located in the mid-low rectum, and 63 (45.7%) patients had a tumor size > 5 cm. Seventy-four (53.6%) patients were downstaged to stage 0-I after NCRT, while 64 patients remained in stage II-III. Eight (5.8%), 17 (12.3%) and 20 (14.5%) patients had positive lymphovascular invasion (LVI), perineural invasion, and tumor deposits, respectively. According to the four-tier AJCC-TRG, 80 (58%) were TRG0-1, while 58 (42%) were TRG2-3. The median CEA, FARI, and PNI values were 3.5 (range 1.8–4.1), 7.7% (range 6.5–8.7), and 45.9 (range 43.2–48.5), respectively. Detailed characteristics of the enrolled patients are shown in Table 1.

**Table 1:** Patient characteristics

Variables	Total number (%)
Gender	
Male	100 (72.5)
Female	38 (27.5)
Age, years [median (IQR)]	60 (53–69)
Site	
Low	43 (31.2)
Middle	75 (54.3)
High	20 (14.5)
Tumor size	
> 5 cm	63 (45.7)
≤ 5 cm	75 (54.3)
cTNM	
II	31 (22.5)
III	107 (77.5)
ypTNM	
0-I	74 (53.6)
II-III	64 (46.4)
Histopathology	
Well differentiation	6 (4.7)
Moderate differentiation	109 (84.5)
Poor differentiation	14 (10.9)
LVI	
Positive	8 (5.8)
Negative	130 (94.2)
PNI	
Positve	17 (12.3)
Negative	121 (87.7)
Tumor deposits	
Positive	20 (14.5)
Negative	118 (85.5)
TRG	
0–1	80 (58.0)
2–3	58 (42.0)
LNH [median (IQR)]	8.8 (5.0–12.0)
CEA [median (IQR)]	3.5 (1.8–4.1)
FARI% [median (IQR)]	7.7 (6.5–8.7)
PNI [median (IQR)]	45.9 (43.2–48.5)
CFP	
Low	95 (68.8)
High	43 (31.2)

*IQR* interquartile rangel, *LVI* lymphovascular invasion, *PNI* perineural invasion, *TRG* tumor regression grade, *LHN* lymph node harvest, *CEA* carcinoembryonic antigen, *PNI* prognostic nutrition index, *FARI* fibrinogen–Albumin Ratio Index, *CFP* CEA-FARI-PNI score

### Optimal cut-off values of CEA, FARI and PNI

**Fig. 2:**
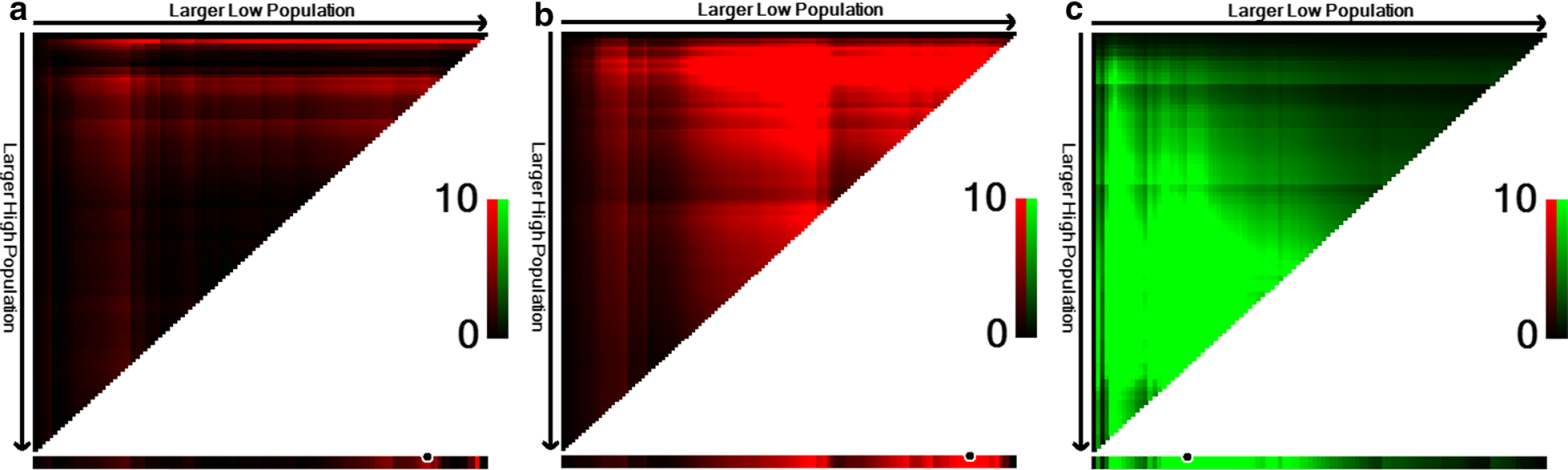
X-tile plot of CEA (a), FARI (**b**), and PNI (**c**). The x-axis of the X-tile plot represented all possible cut-off values for the marker, with the size of the cut-off values increasing from left to right. The brightest pixel (marked by the black circle) represented the optimal cut-off value. Red colouration indicated an indirect association between markers and prognosis, in which green colouration indicated a direct association between markers and prognosis

According to the X-tile program, the optimal cut-off values of OS in CEA, FARI, and PNI were 5.15 ng/ml, 10.56%, and 42.25 g/L, respectively. Detailed data are shown in Fig. 2. Based on these cut-off values, patients were divided into low CEA (≤ 5.15, n = 122), FARI (≤ 10.56, n = 124), PNI (≤ 42.25, n = 27) and high CEA (> 5.15, n = 16), FARI (> 10.56, n = 14), and PNI (> 42.25, n = 111) groups. The CFP scores of CEA, FARI, and PNI were obtained based on the cut-off values of the X-tile program. Likewise, the low (n = 95) and high (n = 43) CFP score groups were also constructed according to the final CEA + FARI + PNI scores.

### Time-dependent ROC analysis of CEA, FARI, PNI, and CFP

**Fig. 3:**
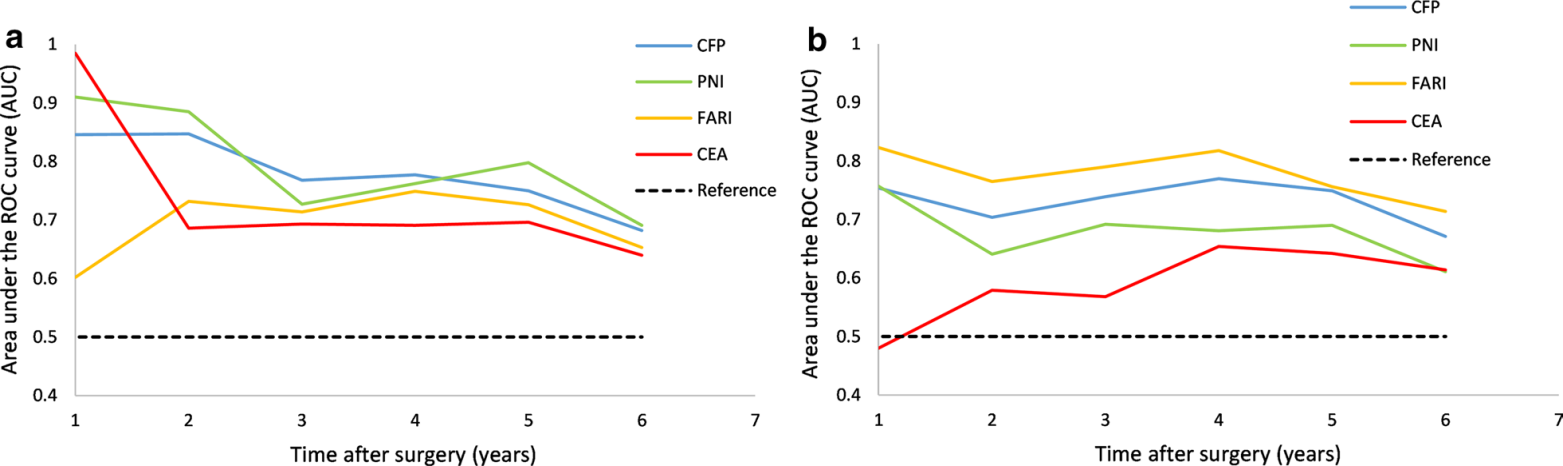
Time-dependent ROC curve. Time-dependent ROC of preoperative CEA, PNI, FARI, and CFP for OS (**a**) and DFS (**b**)

Time-dependent ROC analysis was conducted to compare the ability of the markers to predict OS and DFS. From the second year after surgery, the AUC of CFP for forecasting OS continued to be superior to those of CEA and FARI (Fig. 3a). Meanwhile, the AUC of CFP in forecasting DFS was superior to those of CEA and PNI (Fig. 3b). Unlike CEA, FARI, and PNI, CFP showed a relatively stable ability to predict both OS and DFS. The AUCs of the CFP in predicting 1, 2, 3, 4, 5, and 6 years for OS and DFS were 0.846, 0.847, 0.768, 0.777, 0.75, and 0.682 and 0.754, 0.704, 0.739, 0.77, 0.749, and 0.671, respectively. The data for CEA, FARI, and PNI are shown in Additional file 1: Table S1.

### The correlation between CFP and clinicopathological characteristics

We next analyzed the relationship between CFP and clinicopathological characteristics in LARC patients. The chi-square test showed that the high CFP score group was significantly associate with larger tumor size (p = 0.002), higher ypTNM stage (< 0.001), the presence of perineural invasion (p < 0.001), and poorer tumor response (p = 0.001) compared to the low CFP score group. The CFP score was not significantly correlated with the remaining clinicopathological features, such as sex, age, tumor site, histopathology, total number of lymph nodes harvested (LNH), LVI, and tumor deposits (p > 0.05). The detailed data of the two groups are shown in Table 2.

**Table2:** Characteristics of patients according to CFP score

Variables	Low CFP group	High CFP group	p *value*
Gender			0.729
Male	68 (71.6)	32 (74.4)	
Female	27 (28.4)	11 (25.6)	
Age, years [median (CI)]	60 (58–63)	60 (56–64)	0.899
Site			0.076
Low	35 (36.8)	8 (18.6)	
Middle	46 (48.4)	28 (67.4)	
Upper	14 (14.7)	6 (14.0)	
Tumor size			0.002
> 5 cm	35 (36.8)	28 (65.1)	
≤ 5 cm	60 (63.2)	15 (34.9)	
ypTNM category			< 0.001
0-I	62 (65.3)	12 (27.9)	
II-III	33 (34.7)	31 (72.1)	
Histopathology (N = 129)			0.335
Well differentiation	5 (5.8)	1 (2.3)	
Moderate differentiation	74 (86.0)	35 (81.4)	
Poor differentiation	7 (8.1)	7 (16.3)	
LNH	8.3 (7.3–9.4)	10.0 (8.6–11.5)	0.051
LVI			0.428
Positive	4 (4.2)	4 (9.3)	
Negative	91 (95.8)	39 (90.7)	
PNI			< 0.001
Positve	5 (5.3)	12 (27.9)	
Negative	90 (94.7)	31 (72.1)	
Tumor deposits			0.148
Positive	11 (11.6)	9 (20.9)	
Negative	84 (88.4)	34 (79.1)	
TRG			0.001
0–1	64 (67.4)	16 (37.2)	
2–3	31(32.6)	27 (62.8)	
CEA [median (CI)]	2.6 (2.4–2.9)	5.3 (3.9–7.1)	0.002
FARI, % [median(CI)]	7.0 (6.8–7.3)	9.3 (8.7–9.9)	< 0.001
PNI [median (CI)]	47.4 (46.8–48.0)	42.5 (41.4–43.5)	< 0.001

*CI* confidence interval, *LNH* lymph node harvest, *LVI* lymphovascular invasion, *PNI* perineural invasion, *TRG* tumor regression grade, *CEA* carcinoembryonic antigen, *FARI* Fibrinogen–Albumin Ratio Index, *PNI* prognostic nutritional index

### Survival analysis of CEA, FARI, PNI, and CFP in LARC

**Fig. 4:**
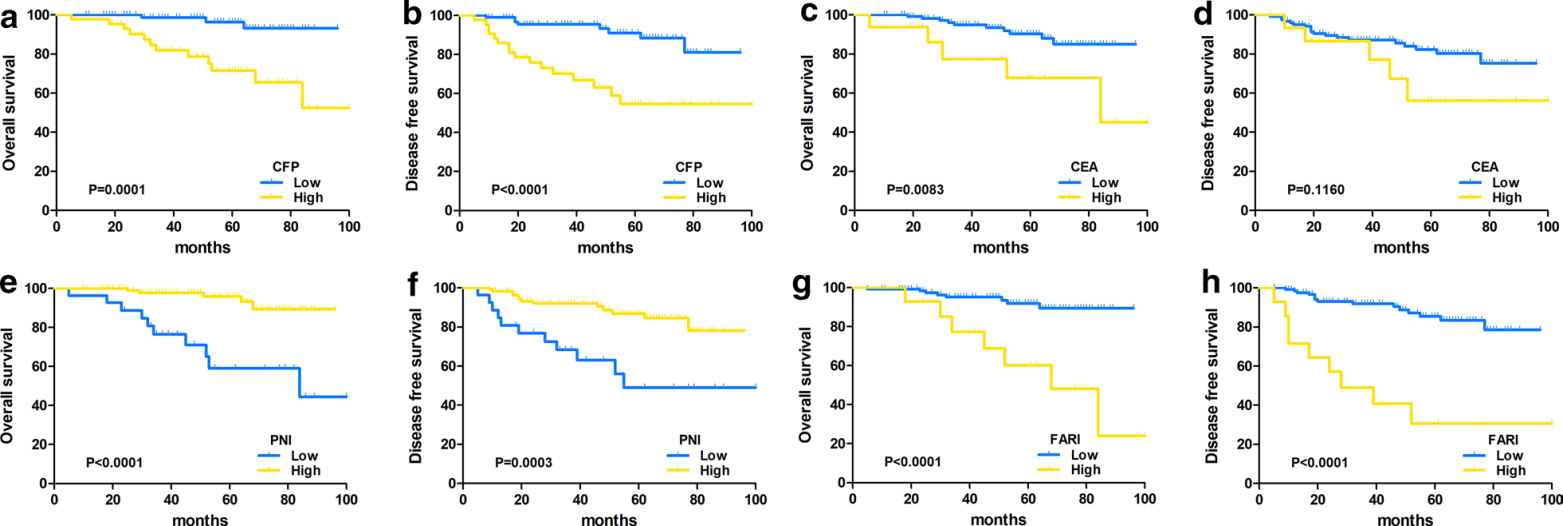
Comparison of OS and DFS between different CEA, FARI, PNI and CFP groups in LARC patients. **a** Kaplan–Meier analysis for OS rate between different CFP groups in LARC patients (p = 0.0001). **b** Kaplan–Meier analysis for the DFS rate between different CFP groups in LARC patients (p < 0.0001). **c** Kaplan–Meier analysis for OS rate between different CEA groups in LARC patients (p = 0.0083). **d** Kaplan–Meier analysis for the DFS rate between different CEA groups in LARC patients (p = 0.1160). **e** Kaplan–Meier analysis for OS rate between different PNI groups in LARC patients (p < 0.0001). **f** Kaplan–Meier analysis for the DFS rate between different PNI groups in LARC patients (p = 0.0003). **g** Kaplan–Meier analysis for OS rate between different FARI groups in LARC patients (p < 0.0001). **h** Kaplan–Meier analysis for the DFS rate between different FARI groups in LARC patients (p < 0.0001).

The follow-up time ranged from 5 to 100 months, and the median follow-up time was 48.5 months. Fifteen (10.9%) patients had died at the last follow-up, and local recurrence with or without metastasis occurred in 24 (17.4%) patients among the 138 eligible patients. According to Kaplan–Meier analysis of OS, CEA (0.0083), FARI (p < 0.0001), PNI (p < 0.0001) and CFP (p = 0.0001) could distinguish patients with poor OS (Fig. 4a, c, e, g), and the cumulative 5-year OS rates of high CEA, high FARI, low PNI, and high CFP were 67.7%, 60.2%, 59.1% and 71.5%, respectively. high FARI (< 0.0001), low PNI (p = 0.0003) and high CFP (< 0.0001) were significantly correlated with poor DFS (Fig. 4b, d, f, h), and the cumulative 5-year DFS rates of high FARI, low PNI, and high CFP were 30.6%, 49.0%, and 54.7%, respectively.

### Univariate and multivariate analysis for OS and DFS

A Cox proportional hazard model was conducted further to demonstrate the prognostic value of the CFP scoring system. Univariate analysis showed that ypTNM stage, the presence of LVI, perineural invasion, tumor deposits, CEA, FARI, PNI, and CFP were significantly associated with OS (Table 3). All potential risk factors (p < 0.1) were adopted for multivariate analysis, and results showed that both a high CFP score (HR = 6.606, p = 0.005) and the presence of LVI (HR = 7.019, p = 0.001) were independent prognostic factors of poor OS in LARC patients undergoing radical surgery following NCRT. As for DFS, univariate analysis showed that tumor size, ypTNM stage, the presence of LVI, perineural invasion, and tumor deposits, FARI, PNI, and CFP were significantly associated with it (Table 3). Multivariate analysis showed that both CFP score (HR = 6.635, p = 0.003), ypTNM stage (HR = 4.785, p = 0.02), perineural invasion (HR = 4.904, p = 0.009), tumor deposits (HR = 7.932, p < 0.001), and FARI (HR = 3.642, p = 0.013) were independent prognostic indicator of DFS in LARC patients undergoing radical surgery following NCRT. (Table 3).

**Table 3:** Univariable and multivariable analyses to determine independent predictors of DFS and OS in LARC patients

	OS	P value		P value	DFS	P value		P value
	Univariate		Multivariate		Univariate		Multivariate	
	HR (95%CI)		HR (95%CI)		HR (95%CI)		HR (95%CI)	
Gender (male vs female)	0.289 (0.064–1.301)	0.106	–	–	0.594 (0.221–1.598)	0.303	–	–
Age, years	1.019 (0.976–1.064)	0.383	–	–	1.006 (0.974–1.039)	0.725	–	–
Tumor site	–	0.736	–	–	-	0.272	–	–
Low vs Upper	0.529 (0.107–2.625)	0.436	–	–	0.447 (0.144–1.398)	0.164	–	–
Middle vs Upper	0.757 (0.205–2.802)	0.677	–	–	0.478 (0.178–1.279)	0.141	–	–
Tumor size (> 5 vs ≤ 5)	3.119 (0.990–9.822)	0.052	–	–	3.819 (1.507–9.679)	0.005	–	–
ypTNM (0-I vs II-III)	5.357 (1.505–19.070)	0.010	–	–	10.853 (3.224–36.529)	< 0.001	4.785 (1.274–17.966)	0.020
LNH	0.999 (0.901–1.107)	0.980	–	–	1.035 (0.957–1.119)	0.385	–	–
LVI (+ vs −)	11.976 (3.712–38.637)	< 0.001	7.019 (2.117–23.267)	0.001	6.990 (2.311–21.140)	0.001	–	–
Perineural invasion (+ vs −)	6.505 (2.340–18.087)	< 0.001	–	–	5.077 (2.210–11.661)	< 0.001	4.904 (1.475–16.301)	0.009
Tumor deposit (+ vs −)	4.476 (1.569–12.768)	0.005	–	–	6.867 (3.061–15.406)	< 0.001	7.932 (2.731–23.038)	< 0.001
CEA (High vs Low)	3.891 (1.316–11.507)	0.014	–	–	2.160 (0.806–5.791)	0.126	–	–
FARI (High vs Low)	6.495 (2.349–17.959)	< 0.001	–	–	7.274 (3.171–16.686)	< 0.001	3.642 (1.316–10.081)	0.013
PNI (Low vs High)	7.764 (2.643–22.810)	< 0.001	–	–	3.922 (1.753–8.773)	0.001	–	–
CFP (High vs Low)	8.117 (2.288–28.789)	0.001	6.606 (1.786–23.705)	0.005	4.994 (2.135–11.682)	< 0.001	6.635 (1.934–22.767)	0.003

*HR* hazard ratio, *CI* cofidence interval, *LNH* lymph node harvest, *LVI* lymphovascular invasion, *PNI* perineural invasion, *CEA* carcinoembryonic antigen, *PNI* prognostic nutritional index, *FARI* Fibrinogen–Albumin Ratio Index, *CFP* CEA-FARI-PNI score

### The relationship between CEA, FARI, PNI, and CFP and response to NCRT

**Fig. 5:**
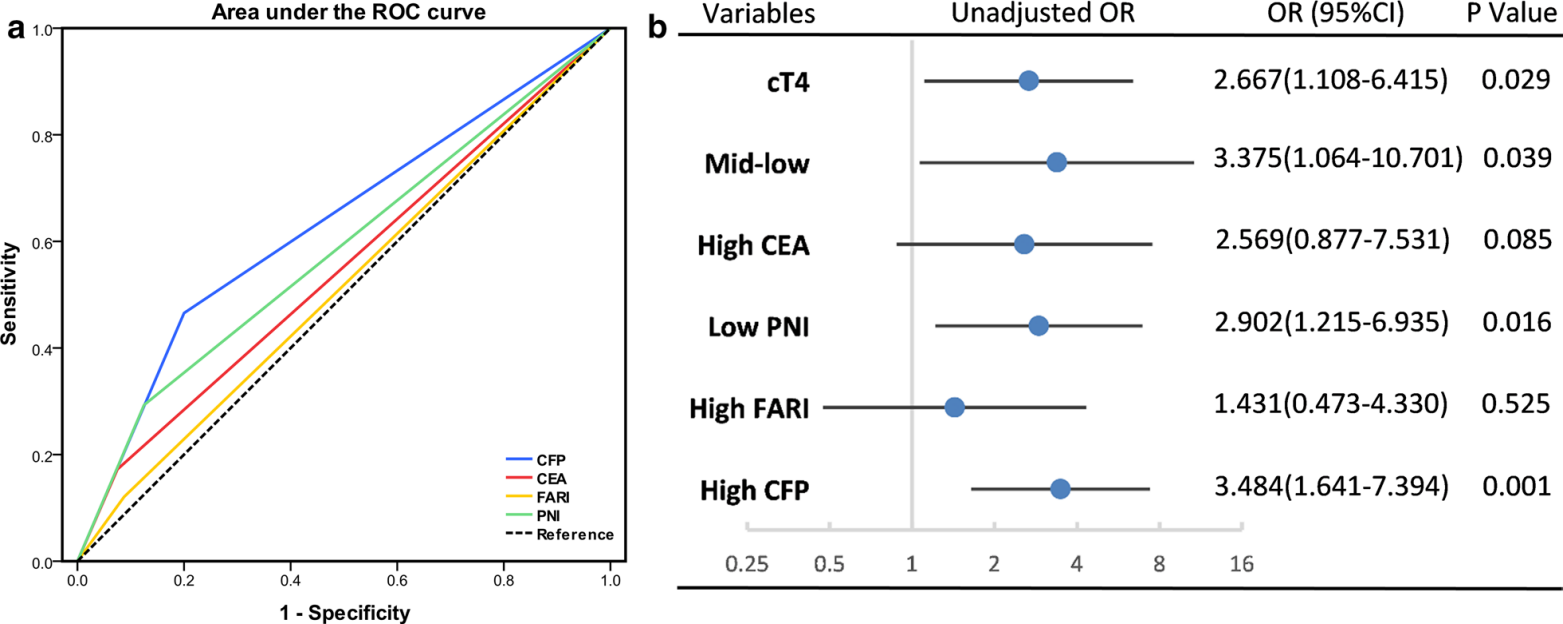
ROC curve and forest plot. **a** ROC curve of CEA, FARI, PNI, and CFP predicts poor response. **b** Forest plot of unadjusted logistic regression to assess the discrimination ability of CEA, FARI, PNI, and CFP for tumor response.

**Fig. 6:**
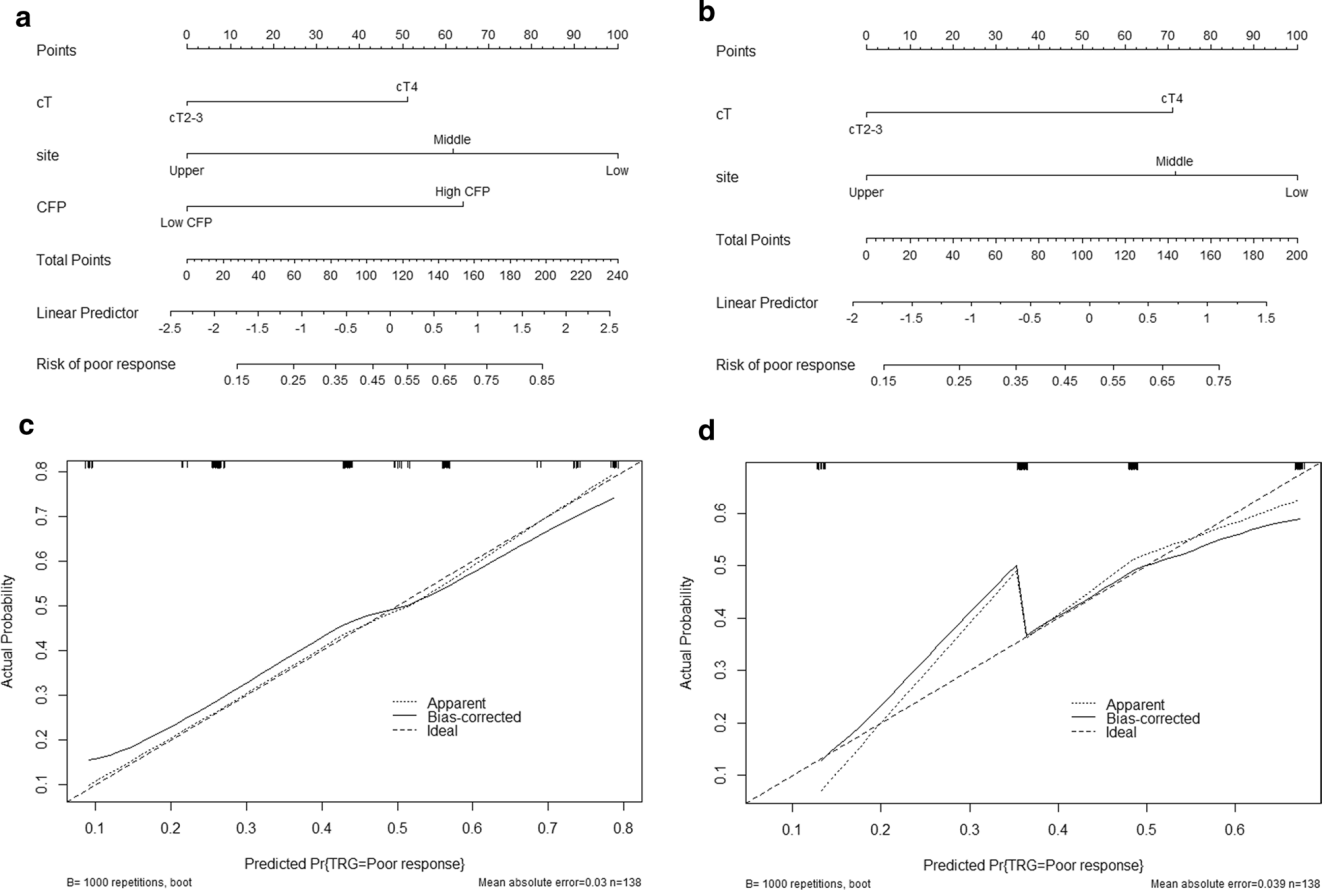
Predicted nomogram and calibration of TRG. Predicted nomogram and calibration of TRG were established by significant factors. **a** Nomogram with CFP score; **b** without CFP score; **c** Calibration with CFP score; **d** without CDP score. The diagonal black dotted line represents a perfect prediction model. The solid black line represents the performance of the nomogram, and a closer fit to the diagonal black dotted line represents a better prediction.

To further explore the clinical utility of CEA, FARI, PNI, and CFP in predicting tumor response to NCRT, ROC curves and logistic regression models were established based on TRG. According to the ROC analysis, the AUC of CFP to predict TRG was 0.633 (p = 0.008), which was superior to those of CEA (AUC = 0.549, p = 0.330), FARI (AUC = 0.517, p = 0.740), and PNI (AUC = 0.584, p = 0.093) (Fig. 5a). In the forest plot of univariate logistic regression analysis, cT4, mid-low tumor site, low PNI, and high CFP were significantly associated with a poor response, while high CEA and high FARI were not (Fig. 5b). In multivariate logistic regression analysis, cT stage (cT4 vs cT2-3, HR = 2.837, p = 0.040), tumor site (lower vs upper, HR = 7.683, p = 0.004; middle vs upper, HR = 3.562, p = 0.058) and CFP (High vs Low, HR = 3.693, p = 0.002) remained significantly associated with TRG. Detailed data are shown in Additional file 1: Table S2. According to the independent risk factors of tumor response that derived from the multivariate logistic regression analysis, we established two nomograms to predict the risk of poor response, one containing CFP, tumor site, cT stage and one only containing tumor site and cT stage. The AUC of the nomogram with CFP (0.717) was better than that without CFP (0.656) (p < 0.05). In addition, the calibration curve of the nomogram with CFP was closer to the ideal curve than that without CFP (Fig. 6c, d).

## Discussion

Rectal cancer is considered a complex disease caused by the interaction of genetic and environmental factors, which also leads to its heterogeneous nature [10]. Although the application of NCRT could shrink the tumor, achieve the objective of downstaging, and reduce the difficulty of surgery and local recurrence rate, the survival of patients is still far from satisfactory. Currently, the high-risk pathological factors for poor prognosis of rectal cancer include poor differentiation, the presence of LVI, perineural invasion, and positive circumferential resection margins. However, these indicators are only available after surgery, limiting their prognostic role in preoperative evaluation. Moreover, the current definition of high-risk factors is clearly inadequate since many patients with high-risk parameters do not have systemic recurrence, while some patients are deemed to be low-risk do. Therefore, the identification of a novel biomarker that could predict prognosis and tumor response is vital. Recently, studies have shown that CEA [7], FARI [20], and PNI [21] are practical predictors of survival and tumor response in LARC patients who underwent radical surgery after NCRT. Hence, we verified the prognostic role of these parameters and established a CFP scoring system. Our study is the first to evaluate the prognostic role of the CFP scoring system in LARC patients, and CFP showed great predictive ability in both survival and tumor response.

Cancer-related inflammation is a defensive response elicited by the body against the tumor, and there is growing evidence that the systemic inflammatory response plays a critical role in the development and progression of malignancy [10]. Combinations of leukocyte-based inflammation markers, such as the neutrophil to lymphocyte ratio, lymphocyte to monocyte ratio, platelet to lymphocyte ratio, and systemic immune-inflammation, have also been reported to be significantly associated with the prognosis of malignant tumors [22–24]. However, NCRT may reduce the total circulating leukocytes and interfere with the inflammatory response of the host, limiting the application of leukocyte-based inflammation biomarkers to predict the prognosis of LARC patients who underwent NCRT. Wang et al. [17] found that both neutrophil-to-lymphocyte, lymphocyte-to-monocyte, platelet-to-lymphocyte and systemic immune-inflammation index (derived from lymphocyte, neutrophil and platelet counts) failed to show an independent prognostic value in patients undertook NCRT. Further, our previous findings were consistent with this point of view [20]. The CFP scoring system is a combination of tumor markers (CEA), inflammatory factors (lymphocytes and fibrinogen), and nutritional factors (albumin). We found that the CFP score based on CEA, FARI and PNI was superior to a single biomarker for precisely predicting the cancer burden and prognosis of the disease for the following reasons. First, lymphocytes, especially CD3^+^ and CD8^+^ T cells, migrate into the tumor microenvironment of LARC patients and play an essential antitumor role. EL Sissy et al. [25] found that the presence of CD3^+^ and CD8^+^ T cells was correlated with survival in LARC patients. Second, the level of circulating fibrinogen is increased by interleukin-6 secreted by tumor cells, and fibrinogen has been found to interact with several growth factors to induce tumor seeding and promote the invasion of tumor cells, leading to a poor prognosis [26]. Third, poor nutritional status is reflected by circulating albumin, which promotes IL-1, IL-6, TNF-α, and acute-phase reactant release, increasing the morbidity and mortality of patients [27].

In our study, CEA, fibrinogen, albumin, and the total lymphocyte count were routine indicators examined before curative surgery, as well as FARI and PNI were the combinations of some of these indicators, making these biomarkers inexpensive and clinically practical. We found that high FARI, low PNI, and a CFP score of 1 were significantly associated with poor DFS and OS. CEA is also closely related to OS, but for DFS, there is only a tendency for a high CEA level to predict a poor DFS. The time-dependent ROC curve indicated that CFP has stable predictive performance in both OS and DFS for each time period and is an independent prognostic risk factor for both OS (HR = 6.606, p = 0.005) and DFS (HR = 6.635, p = 0.003), suggesting that the novel CFP score was an appropriate biomarker for forecasting survival in LARC patients who underwent TME following NCRT. Recently, some researchers found that tumor budding, inflammatory infiltration and redox status could predict the prognosis of colorectal cancer and leads to new prognostic subgroups [28–30]. Some researchers found that clinic-genetic profile, which contain a complete summary of the patient status, and Raman-enhanced spectroscopy probe providing new possibilities in personalized medicine and prognostic views in cancer patient [31, 32]. In our future study, we will combine these promising findings with CFP to predict prognosis and tumor response among LARC patients.

The TRG scoring system provides a clinically useful indicator of tumor response to chemoradiotherapy and guides subsequent adjuvant treatment. Patients who achieve PCR do not need adjuvant therapy. Various TRG scoring systems exist, including quantitative and semiquantitative scoring systems, to grade the ratio between fiber and residual tumor cells [33–35]. By comparing the four most commonly used TRG systems, Trakarnsanga et al. [36] found that AJCC-TRG was the most accurate. These TRG systems can indeed predict improved DFS and OS [37], but TRG can only be obtained after surgical resection and cannot be used for prediction before surgery. Currently, rectal cancer patients who achieve a clinical complete response can use a watch and wait approach to avoid a series of complications and the associated risk of perioperative death caused by the TME procedure. Post-NCRT examinations such as digital rectal examination, endorectal ultrasonography, and magnetic resonance imaging (MRI) were used to determine the clinical complete response of LARC patients [38]. However, Liu et al. [39] performed the aforementioned examinations on 124 rectal cancer patients who underwent NCRT and found that although mucosal integrity, endorectal ultrasound, and MRI had a high specificity (94.23, 93.90, and 93.27%, respectively) for predicting complete response, their sensitivity was only 25%. In addition, blood-based biomarkers such as circulating tumor DNA [40] and the modified Glasgow prognostic score [13] were associated with tumor response. However, these indicators were not routinely tested during treatment, possibly limiting their utility. Therefore, we further explored the association between CFP and NCRT outcomes, and our findings showed that the AUC (0.633) of CFP was superior to CEA (0.549), FARI (0.517), and PNI (0.584). Multivariate analysis indicated that a high CFP score (HR = 3.693, p = 0.002) was an independent risk factor for poor tumor response (TRG2-3). We combined the clinical T stage, tumor site, and CFP to establish a nomogram that predicted the probability of poor response, and the AUC was 0.717, which was better than the AUC (0.656) without CFP (p < 0.05), suggesting that CFP is a reliable predictor for TRG.

However, some limitations exist in this study. First, this is a retrospective study, so some selection bias inevitably exists. Second, the sample size of this study is relatively small, reflecting the difficulties of subgroup analysis, and external validation of the existing results is lacking. In the future, more patients should be included, and the follow-up time should be extended to further verify these findings. In summary, this study is the first to construct a CEA-FARI-PNI score and to investigate the predictive role of survival and chemoradiotherapy outcome in CEA, FARI, PNI, and CFP scores. The CFP score is a biomarker routinely measured in clinical practice and is an available and promising biomarker for predicting not only prognosis but also chemoradiotherapy outcome in LARC patients who underwent radical surgery after NCRT.

## Conclusion

In summary, our findings indicate that the CFP score is an effective and independent prognostic factor of OS and DFS for patients with LARC undergoing NCRT, and it could also effectively predict the tumor response.

## Supplementary Information

**Additional file 1: Figure S1.** Flowchart of eligible cases selection.

**Additional file 2: Table S1.** AUC values of CEA, FARI, PNI and CFP.

**Additional file 3: Table S2.** Multivariate logistic regression analysis for TRG in LARC patients.

## Data Availability

The data that support the findings of this study are available on request from the corresponding author. The data are not publicly available due to privacy or ethical restrictions.
