# Supplementary figures and images for: High CFP score indicates poor prognosis and chemoradiotherapy response in LARC patients
Source: Cancer Cell Int. 2021 Apr 13;21:205. doi: 10.1186/s12935-021-01903-1 (PMC8045186; doi:10.1186/s12935-021-01903-1)

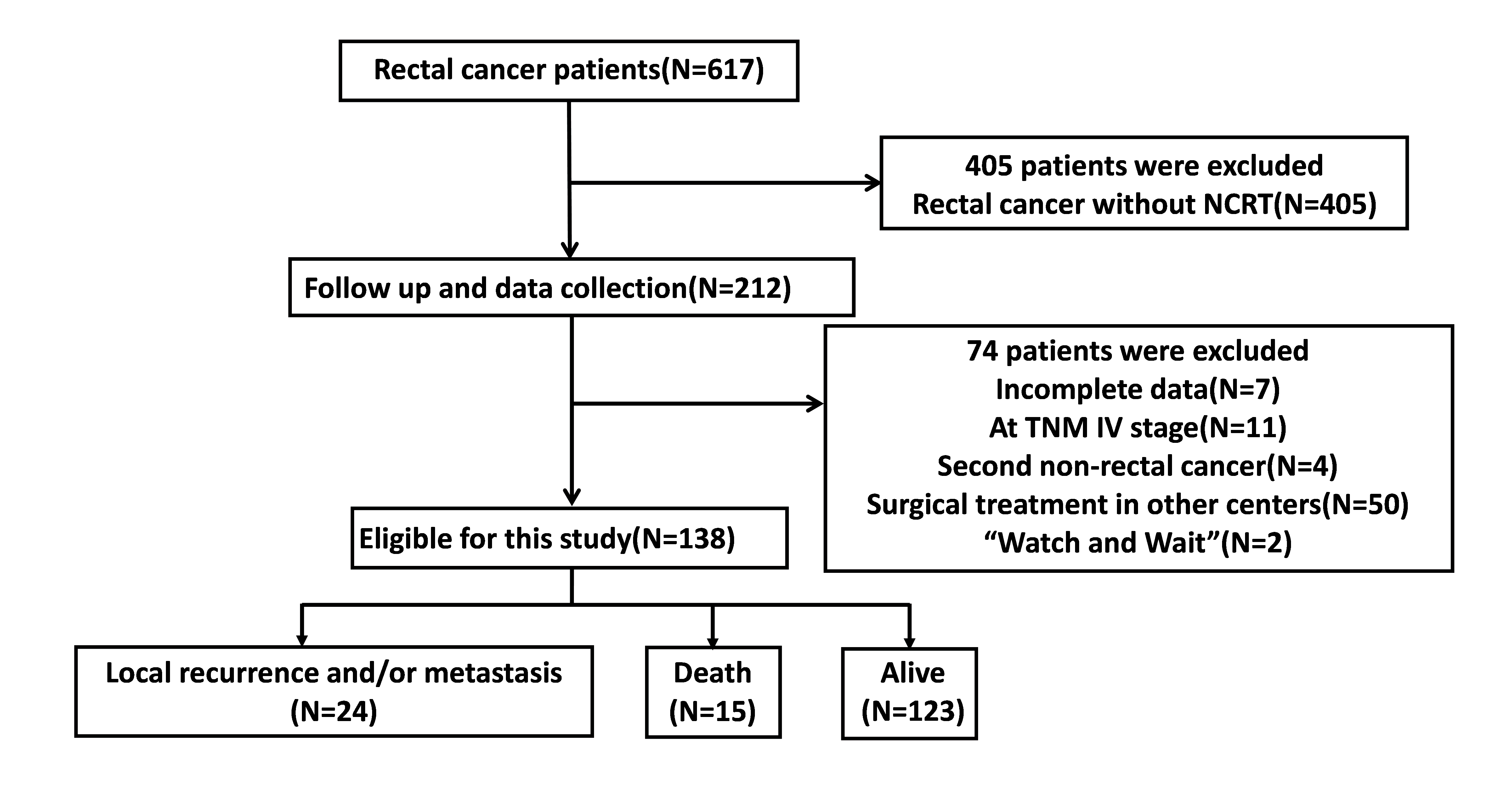

Supplement: Supplementary file 1 — Additional file 1: Figure S1. Flowchart of eligible cases selection. [file 12935_2021_1903_MOESM1_ESM.tif]
