# Supplementary material for: High CFP score indicates poor prognosis and chemoradiotherapy response in LARC patients
Source: Cancer Cell Int. 2021 Apr 13;21:205. doi: 10.1186/s12935-021-01903-1 (PMC8045186; doi:10.1186/s12935-021-01903-1)
Supplement: Supplementary file 2 — Additional file 2: Table S1. AUC values of CEA, FARI, PNI and CFP. [file 12935_2021_1903_MOESM2_ESM.docx]

**Supplementary Table 1**

| OS/AUC | CEA | FARI | PNI | CFP |
| --- | --- | --- | --- | --- |
| 1 | 0.985 | 0.602 | 0.91 | 0.846 |
| 2 | 0.686 | 0.732 | 0.885 | 0.847 |
| 3 | 0.693 | 0.714 | 0.727 | 0.768 |
| 4 | 0.691 | 0.749 | 0.762 | 0.777 |
| 5 | 0.696 | 0.726 | 0.798 | 0.75 |
| 6 | 0.64 | 0.653 | 0.691 | 0.682 |
| DFS/AUC |  |  |  |  |
| 1 | 0.48 | 0.823 | 0.757 | 0.754 |
| 2 | 0.579 | 0.765 | 0.641 | 0.704 |
| 3 | 0.568 | 0.79 | 0.692 | 0.739 |
| 4 | 0.654 | 0.818 | 0.681 | 0.77 |
| 5 | 0.642 | 0.756 | 0.69 | 0.749 |
| 6 | 0.614 | 0.714 | 0.611 | 0.671 |

AUC: area under receiver operating characteristics curve, CEA: carcinoembryonic antigen, FARI: fibrinogen–Albumin Ratio Index, PNI: prognostic nutrition index, CFP: CEA-FARI-PNI score
