# Supplementary material for: High CFP score indicates poor prognosis and chemoradiotherapy response in LARC patients
Source: Cancer Cell Int. 2021 Apr 13;21:205. doi: 10.1186/s12935-021-01903-1 (PMC8045186; doi:10.1186/s12935-021-01903-1)
Supplement: Supplementary file 3 — Additional file 3: Table S2. Multivariate logistic regression analysis for TRG in LARC patients. [file 12935_2021_1903_MOESM3_ESM.docx]

**Supplementary Table S2.** Multivariate logistic regression analysis for TRG LARC patients

| **Variables** | **Score** | **N** |  |  |
| --- | --- | --- | --- | --- |
|  | | | **Multivariate** |  |
|  | | | **OR (95%CI)^a^** | **P *value*** |
| **cT** | **cT2-3** | **112** | **1 (-)** | **-** |
|  | **cT4** | **26** | **2.837 (1.046-7.694)** | **0.040** |
| **Site** | **Upper** | **20** | **1 (-)** | **0.014** |
|  | **Low** | **43** | **7.683 (1.882-31.356)** | **0.004** |
|  | **Middle** | **75** | **3.562 (0.956-12.994)** | **0.058** |
| **CFP** | **Low** | **95** | **1 (-)** | **-** |
|  | **High** | **43** | **3.693 (1.615-8.441)** | **0.002** |

OR: odds ratio, CI: confidence interval, CFP: CEA-FARI-PNI score

^a^ Adjusted by CEA and PNI
